# Long-Term Visual Training Increases Visual Acuity and Long-Term Monocular Deprivation Promotes Ocular Dominance Plasticity in Adult Standard Cage-Raised Mice

**DOI:** 10.1523/ENEURO.0289-17.2017

**Published:** 2018-01-18

**Authors:** Leon Hosang, Rashad Yusifov, Siegrid Löwel

**Affiliations:** 1 Department of Systems Neuroscience, J.F.B. Institut für Zoologie und Anthropologie, Universität Göttingen, Göttingen, D-37075, Germany; 2 Göttingen Graduate School of Neurosciences, Biophysics and Molecular Biosciences (GGNB), Göttingen, D-37077, Germany; 3 Sensory Collaborative Research Center 889, University of Göttingen, D-37075 Göttingen, Germany

**Keywords:** cortical plasticity, intrinsic signal optical imaging, mouse vision, optomotry, visual water task

## Abstract

For routine behavioral tasks, mice predominantly rely on olfactory cues and tactile information. In contrast, their visual capabilities appear rather restricted, raising the question whether they can improve if vision gets more behaviorally relevant. We therefore performed long-term training using the visual water task (VWT): adult standard cage (SC)-raised mice were trained to swim toward a rewarded grating stimulus so that using visual information avoided excessive swimming toward nonrewarded stimuli. Indeed, and in contrast to old mice raised in a generally enriched environment (Greifzu et al., 2016), long-term VWT training increased visual acuity (VA) on average by more than 30% to 0.82 cycles per degree (cyc/deg). In an individual animal, VA even increased to 1.49 cyc/deg, i.e., beyond the rat range of VAs. Since visual experience enhances the spatial frequency threshold of the optomotor (OPT) reflex of the open eye after monocular deprivation (MD), we also quantified monocular vision after VWT training. Monocular VA did not increase reliably, and eye reopening did not initiate a decline to pre-MD values as observed by optomotry; VA values rather increased by continued VWT training. Thus, optomotry and VWT measure different parameters of mouse spatial vision. Finally, we tested whether long-term MD induced ocular dominance (OD) plasticity in the visual cortex of adult [postnatal day (P)162–P182] SC-raised mice. This was indeed the case: 40–50 days of MD induced OD shifts toward the open eye in both VWT-trained and, surprisingly, also in age-matched mice without VWT training. These data indicate that (1) long-term VWT training increases adult mouse VA, and (2) long-term MD induces OD shifts also in adult SC-raised mice.

## Significance Statement

Usually, mice predominantly rely on olfactory and tactile cues. We here show that visual capabilities of mice can markedly improve if vision becomes more behaviorally relevant: long-term vision training in the visual water task (VWT) increased mouse visual acuity (VA) by >30%. Moreover, a direct comparison of VWT-determined VA with optomotry-determined spatial frequency threshold of the optomotor (OPT) response revealed that these two behavioral tests measure different parameters of mouse spatial vision. Finally, we report that long-term monocular deprivation (MD) could induce ocular dominance (OD) plasticity in the visual cortex of old standard cage (SC)-raised mice. Overall, our data suggest that long-term changes in sensory input can boost sensory processing and induce plastic changes even at an advanced age.

## Introduction

In everyday life, mice predominantly rely on olfactory cues and tactile information, partially owing to their nocturnal biorhythm. In contrast, mouse visual acuity (VA) is low compared to more visual animals like cat and squirrel or the rhesus monkey, raising the question whether mouse visual capabilities can improve if vision gets more behaviorally relevant. The visual water task (VWT; Prusky et al., 2000b) is a visual discrimination task based on reinforcement learning. In this task, mice are released into a water-filled tank and have to choose between two paths, each ending in front of a monitor on which visual stimuli are displayed. For testing grating acuity, animals were trained to swim toward the monitor displaying a sine wave grating while equiluminant gray was the nonrewarded stimulus. A submerged escape platform in front of the grating stimulus served as reward. Thus, using visual information for the behavioral decision avoided excessive swimming toward nonrewarded stimuli. Using the VWT, mice reached average visual acuities of 0.49 cycles per degree (cyc/deg), whereas rat VA was 0.94 cyc/deg, approximately twice that of mice (Prusky et al., 2000b).

If mice were raised in standard cages (SCs) with opaque walls this lack of visual stimuli resulted in a decreased VA in mice (Prusky et al., 2000a). Raising mice in a so-called “enriched environment” with, e.g., more animals housed together, more space, labyrinths and running wheels, did, however, not obviously increase VA of old mice beyond the values observed in mice raised in conventional SCs with translucent lids (Prusky et al., 2000a; Greifzu et al., 2016). Given these observations, we were interested in examining whether increasing the behavioral importance of visual stimuli would increase mouse visual capabilities. To address this question, we performed long-term VWT training until values reached a plateau. Indeed, extended vision training of mice strongly increased mouse VA even into the rat range of VAs.

It has previously been documented that the spatial frequency threshold of the optomotor (OPT) reflex of the open eye increases after few days of monocular deprivation (MD; Prusky et al., 2006). Daily testing of mice in the virtual-reality OPT system leads to a maximal enhancement of OPT thresholds compared to nontrained mice: after 7 d of MD, thresholds increased by 25-30% compared to baseline values before MD; on reopening the deprived eye, thresholds again returned to lower binocular values as measured before MD (Prusky et al., 2006). Since spatial frequency thresholds determined by optomotry, even those after MD and daily testing, are lower than VAs measured with the VWT, we wanted to examine whether monocular testing could also increase VA when assessed by the VWT. While individual mice responded differently to MD, average monocular VA in the VWT did not increase after MD; additionally, eye reopening did not initiate a decline of thresholds to pre-MD binocular values as observed by optomotry. Instead, values increased by continued VWT training.

In SC-raised mice, ocular dominance (OD) plasticity is age dependent: in postnatal day (P)25–P35 mice, 4 d of MD are sufficient to induce OD shifts toward the open eye; thereafter, 7 d of MD are needed. Beyond P110, even 14 d MD failed to induce OD plasticity in mouse V1 (Lehmann and Löwel, 2008; Espinosa and Stryker, 2012; Levelt and Hübener, 2012). In contrast, various environmental manipulations have been shown to prolong the sensitive phase for OD plasticity into adulthood, e.g., previous MD in young animals (Hofer et al., 2006), environmental enrichment (Sale et al., 2007; Greifzu et al., 2014), voluntary physical exercise (Kalogeraki et al., 2014), dark exposure (He et al., 2006; Stodieck et al., 2014), and forced visual stimulation (Matthies et al., 2013). Thus, OD plasticity is principally possible beyond P110, raising the question whether the previously tested MD duration of 14 d was just not long enough to induce network changes. We therefore tested whether longer-term monocular visual experience and a higher behavioral importance of the visual stimuli by VWT training is necessary to induce OD shifts after MD in adult SC-raised mice. Indeed, MD of at least 39 d induced OD plasticity in adult (P162–P182) VWT-trained mice. Surprisingly, however, long-term MD also induced OD plasticity in control age-matched (P177–P180) non-VWT-trained animals, indicating that MD duration, not VWT training, was the crucial parameter for the successful induction of OD plasticity. 

## Materials and Methods

### Animals

All experimental procedures were approved by the local government (Niedersächsisches Landesamt für Verbraucherschutz und Lebensmittelsicherheit). The experimental procedures comply with National Institutes of Health guidelines for the use of animals. We used a total of 26 male C57BL/6J mice; all animals descended from the mouse colony at the central animal facility of the University Medical Center Göttingen and were raised in standard translucent cages with an open grid cover and wood chip bedding (32 × 16 × 14 cm, three to five animals/cage, individual housing during MD), at a 12/12 h light/dark cycle with food and water provided ad libitum.

### Schematic experimental design

**Figure 1.**
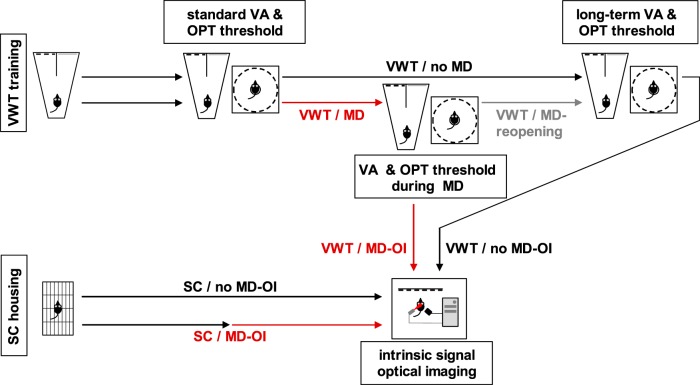
Scheme of the experimental design. Two independent cohorts of mice were trained daily in the visual water task (VWT) until reaching binocular visual acuity (VA) based on established protocols (Prusky et al., 2000b, 2004). At the day of standard VA assessment, the two groups were also tested in the virtual-reality optomotor (OPT) system (optomotry) to determine the spatial frequency threshold of the OPT response (standard OPT threshold). One of the cohorts then obtained monocular deprivation (MD). Both cohorts were continued to be trained in the VWT. The monocular VA and OPT threshold of the VWT/MD group (during MD) was again determined after reaching the criterion (see Materials and Methods). In about half of the mice of the VWT/MD group, the MD was reopened and mice were continued to be trained daily in the VWT (VWT/MD-reopening). The binocular long-term VA and OPT threshold of both the VWT/no-MD and VWT/MD-reopening group was again tested after a comparable training time (see Materials and Methods). Instead of reopening the MD, optical imaging of intrinsic signals (OI) was performed with the other half of the deprived group (VWT/MD-OI). For comparison, a cohort of mice that had been deprived for a comparably long time but not VWT-trained was imaged (SC/MD-OI). As a control for the long-term trained VWT/MD group, we used the VWT/no-MD group after finishing the VWT (VWT/no-MD-OI). Likewise, we also tested an age-matched nondeprived, SC-housed cohort (SC/no-MD-OI).

Figure 1 schematically depicts the experimental design, which is described in detail in the following paragraphs.

### Visual water task (VWT)

#### Setup

Visual acuity (VA) of all experimental animals was measured using the VWT (Prusky and Douglas, 2004). The VWT is a reward-based behavioral test developed to determine visual capabilities of rodents. A trapezoidal steel tank measuring 118 (length) × 40 (height) × 80 (width at wide end) × 25 (width at narrow end) cm serves as basin for the mice. The wide end part is made of transparent acrylic glass with two monitors (35 × 26 cm) facing the tank’s inner and positioned in 15 cm height. The monitors are connected to a MacPro (Apple). The tank can be fully or partially divided along its length via steel plates (40 × 117.5, 45.5, and 26.5 cm for full, medium, or short divider, respectively), thereby creating two paths, each of which ends in front of a monitor. The choice line is defined by the lengths of the dividers. The tank is filled with shallow tepid water to a height of 15 cm which is changed daily. A submerged transparent escape platform [33 (length) × 12.8 (width) × 13.5 (height) cm] is positioned in front of the monitor displaying the “rewarded” visual stimulus, in our experiments, a sinusoidal vertical black and white grating at 100% contrast, presented at variable spatial frequencies ranging from 0.086 cyc/deg to maximally 1.52 cyc/deg. Isoluminent gray was displayed on the reference screen. The software Vista (CerebralMechanics) was used to control the stimulus and record the temporal course of the experiment. We ensured that luminance of visual stimuli on the two monitors was identical. The rewarded stimulus was displayed either on the left (L) or right (R) screen based on a repetitive pseudo-random pattern (LRLRRLRLL). The narrow end of the basin serves as release chute for the mice. Upon reaching the platform, the mice are transferred to a box and placed on a heating pad until the next round. Choosing the wrong path results in negative reinforcement since the mice have to swim back and around the divider to reach the platform.

#### Pretraining

In the pretraining phase, mice learned to swim toward the screen showing the rewarded stimulus at the baseline spatial frequency (0.086 cyc/deg) in a fully divided tank, without the reference screen option. Each mouse had to perform the task five times on the first day, 10 times on the second, and 15 times on the third day.

#### Training

In the subsequent training phase, the mice learned to discriminate the rewarded stimulus from isoluminant gray. The rewarded stimulus was initially presented at baseline spatial frequency on either the left or right screen following a pseudo-random procedure. The short divider was used on the first 3 d before switching to the medium length divider used during the remaining training and the entire subsequent testing phase. The animals had to perform two times 15 trials daily. In between, mice were allowed to recover for 90 min. In case an animal developed a tendency to swim either always left or right, a pattern displaying the stimulus twice as often at the nonbiased side was used to counteract false learning. If a mouse achieved accuracy of 80-100% in four to five consecutive blocks of 10 trials, it was conveyed to the test phase.

#### Test Phase I: standard binocular visual acuity (VA)

Standard VA was determined according to the original protocol of Prusky et al. (2000b). In general, a VA threshold was attained when the animal failed to achieve 70% accuracy at a particular spatial frequency.

Again, mice were tested twice daily (*n* = 16, divided in two groups; see below) as described above. Varying criteria at different spatial frequency ranges were applied for successfully attaining a particular threshold (Table 1). Spatial frequencies were adjusted individually for each animal based on its progress. On passing the criterion for a given frequency, the spatial frequency was increased by one step. Otherwise, failing the criterion was considered a “break”: the spatial frequency of the visual stimulus was decreased by three steps before the test phase was continued. When a mouse had three such breaks at very similar frequency steps, the spatial frequencies one step below these three breaks (highest spatial frequency at which the mouse could still achieve at least 70% accuracy) were averaged and served as a measure for its “standard VA.”

**Table 1.** Rules underlying the VWT Test Phases I–III

	Visual water task - rules		
Frequency range (cyc/deg)	0.086–0.172	0.201–0.372	≥0.401
Criteria to pass(correct trials/total trials)	1/1	3/3	5/5
	or 3/4	or 5/6	or 7/10
	or 7/10	or 7/10	
	→ break	→ break	→ break

Different criteria applied for different spatial frequency ranges, e.g., in the lowest range, one correct trial was sufficient for passing this frequency step, resulting in an increase of the spatial frequency. If an animal failed the first trial, it still had the chance to pass by correctly choosing in three out of four or seven out of 10 trials. If it failed, the spatial frequency was decreased by three steps. In higher frequency ranges, the criteria were more strict to exclude coincidental passing.

#### Test Phase II: visual acuity (VA) during monocular deprivation (MD)

To test whether monocular visual experience increased spatial frequency thresholds also in the VWT, as previously described for optomotry (Prusky et al., 2004), one group of the VWT-tested mice (VWT/MD group, *n* = 11/16) obtained an MD (for details, see below) after another two breaks in the same frequency range to ensure stability of the assessed VA. Test Phase II started on the day after MD.

The first 7 d of Phase II in the MD mice were considered as an adaption phase (Adaption Phase I) for getting used to monocular swimming since our measurements showed that most (82%) of the mice displayed strong reductions in monocular VA immediately after MD compared to their binocular VA values. Any breaks during this adaptation phase did not enter final analyses. Monocular VA during MD was calculated as described for Test Phase I.

The remaining mice (VWT/no-MD group, *n* = 5/16) were not monocularly deprived and continued to be tested binocularly as in Phase I (no Test Phase II). We ensured that the amount of test days of this group was similar to the VWT/MD-reopening subgroup.

#### Test Phase III: long-term visual acuity (VA)

Again, the animals of the VWT/MD group were tested until they had a total of five breaks at similar spatial frequencies to ensure stability of the values. Then, the deprived eyes of 6 of the 11 animals of the MD group were reopened (VWT/MD-reopening subgroup) to test whether restoration of binocular vision would increase VA compared to Test Phase II. In the remaining VWT-MD animals (VWT/MD-OI subgroup; *n* = 5/11), the MD eye remained closed until V1 activity was visualized using intrinsic signal optical imaging.

Test Phase III started on the day after reopening the MD eye. As in Test Phase II, any breaks during the next 7 d (Adaption Phase II) did not enter final analyses. Testing of both the VWT/MD-reopening and VWT/no-MD (sub)groups continued until all mice had reached five breaks at similar spatial frequency values, i.e., VA did not increase any further and reached a plateau. The three highest values of these last five breaks served as the basis to calculate the “long-term VA.”

The total testing time (in days) of the VWT/no-MD group and the VWT/MD-reopening subgroup until the determination of long-term VA was similar.

### Virtual-reality optomotor (OPT) system

We used the virtual-reality OPT system to additionally determine basic parameters of spatial vision in all experimental mice also performing the VWT training (*n* = 17), such as the spatial frequency threshold of the OPT reflex (Prusky et al., 2004). 

Briefly, freely moving mice were placed on a small platform surrounded by four flat screen monitors (33.5 × 26.5 cm) forming the walls of a box. Ceiling and floor of the box are made of mirrors, creating the impression to the animal that it was sitting in an endless cylinder. A rotating sine wave grating was projected on the screens, creating the illusion of a virtual cylinder moving around the mouse. The mouse behavior could be followed via a camera attached to the lid of the box, allowing to center the virtual cylinder on the animal´s eyes and track its head movements. In the mouse, the OPT reflex is triggered only by stimuli moving in a temporal-to-nasal direction, and manifests itself as smooth tracking movement of head and trunk in the direction of the moving stimulus. The spatial frequency of the grating stimulus was increased until the reflex was no longer elicited. The highest spatial frequency stimulus eliciting an OPT response at full contrast was taken as the spatial frequency threshold. The MD group was measured at three different time points corresponding to VA assessments in the VWT: after three stable breaks before MD (standard), before MD reopening, and at the end of the VWT training (long-term).

The group without MD was measured at two different time points, again corresponding to VA assessments in the VWT: after three stable breaks in the VWT (standard) and at the end of the VWT training (long-term).

### Monocular deprivation (MD)

MD was always performed on the right eye, according to published protocols (Gordon and Stryker, 1996; Cang et al., 2005). Anesthesia was provided via inhalation of 2% isoflurane in a 1:1 mixture of nitrous oxide (N_2_O) and oxygen (O_2_), and maintained at 1.5% isoflurane. Eyelids of the right eye were trimmed and treated with an antibiotic gel (gentamicin). Upper and lower eyelids were closed with two mattress stitches fixed with a surgical knot. Mice were returned to their home cages and checked daily to ensure that the eyes remained closed.

### Visualizing ocular dominance (OD) plasticity with intrinsic signal optical imaging (OI)

In another set of experiments, we visualized V1 responses in long-term VWT-trained mice with long-term MD (VWT/MD-OI subgroup, *n* = 5/11) using intrinsic signal optical imaging (Cang et al., 2005). Imaging of VWT-trained mice without MD (VWT/no-MD-OI group, *n* = 4/5; after finishing the VWT) served as control. In addition, two groups of age-matched long-term MD (SC/MD-OI, *n* = 4) and no-MD (SC/no-MD-OI, *n* = 5) SC-raised mice that did not experience long-term VWT training were used for comparison.

Briefly, mice were box-anesthetized with 2% halothane in a 1:1 mixture of O_2_/N_2_O and injected with atropine (0.1 mg/mouse, s.c.; Franz Köhler), dexamethasone (0.2 mg/mouse, s.c.; Ratiopharm), and chlorprothixene (0.2 mg/mouse, i.m.; Sigma). After stereotactically holding the mice, anesthesia was maintained at 0.8% halothane in O_2_/N_2_O (1:1). After an incision of the skin above the visual cortex, low-melting agarose and a glass coverslip were placed on the skull above the exposed visual cortical area.

#### Data acquisition and visual stimulation

Mouse V1 responses were recorded through the skull using the Fourier imaging technique developed by Kalatsky and Stryker (2003) and optimized for OD-plasticity assessment by Cang et al. (2005). V1 responses were visualized using a CCD camera (Dalsa 1M30) with a 135 × 50 mm tandem lens configuration (Nikon) and red illumination light (610 ± 10 nm). The light absorption spectrum of oxygenated hemoglobin differs from that of deoxygenated hemoglobin. When illuminated with red light of 610 ± 10 nm wavelength, the light absorption of deoxygenated hemoglobin is higher than that of oxygenated hemoglobin. This results in a decrease in light reflectance of active cortical regions (Grinvald et al., 1986), so that active brain regions appear darker in the images. Frames were acquired at 30 Hz, temporally binned to 7.5 Hz and stored as 512 × 512 images after spatial binning of the camera image. A high refresh rate monitor (Hitachi, ACCUVUE, HM-4921-D, 21″) positioned 25 cm in front of the mouse eyes displayed the visual stimuli. Stimuli consisted of white drifting horizontal bars (width 2°), presented at 0.125 Hz on black background (100% contrast). To calculate OD, the stimulus was restricted to the binocular visual field of the left V1 (-5° to +15° azimuth with 0° corresponding to the frontal midline). The mice were alternately stimulated through the left and right eye by covering the other eye.

#### Data analyses

The acquired frames were used to calculate visual cortical maps by extracting the signal at the stimulus frequency via Fourier analysis (custom software by Kalatsky and Stryker, 2003): The phase component represents the retinotopy, the amplitude component represents the intensity of cortical activation expressed as the fractional change in reflectance x10^4^ and was used for the calculation of OD (Cang et al., 2005). At least three maps per animal were averaged to calculate the OD index (ODI) as follows: (C − I)/(C + I), with C and I representing the response magnitudes to each pixel to visual stimulation of the contralateral and ipsilateral eye, respectively. The ODI ranges from −1 to +1 with negative and positive values corresponding to ipsilateral and contralateral values, respectively.

### Statistical analysis

Table 2 contains information about groups, tested parameter, number of animals, lower and upper 95% confidence interval of the means, data structure (distribution), (sub)group comparisons, types of tests used, and statistical readout. No animals were removed from the analyses. Gaussian distribution was tested by Kolmogorov–Smirnov tests with Lilliefors´ correction. In case of normal distribution, intra- and intergroup comparisons were analyzed by two-tailed paired or unpaired *t* tests. In some cases where the Kolmogorov–Smirnov test with Lilliefors’ correction could not be applied due to a low number of animals, intergroup comparisons were analyzed using the nonparametric Mann–Whitney test. The levels of significance were set as *p < 0.05; **p < 0.01; ***p < 0.001. Visual capabilities (VA, OPT threshold) and imaging parameters (ODI, V1 activation) are represented as mean ± SEM. Ages and training time (trials, days) are represented as mean ± SD.

**Table 2.** Statistical analysis

**Figure**	**Group**	**Parameter**	***n***	**CI_95_**	**Data structure**	**Comparison**	**Type of test**	***p*/*r*^2^ value**
5	VWT/no-MD and VWT/MD-reopening	[A] no-MD – VA % gain[B] MD – VA % gain	56	[9.692; 53.92][11.10; 57.88]	Normal distributionNormal distribution	[A] vs [B]	Unpaired *t* test	*p* = 0.8328
6*A*	VWT/no-MD	[A] standard VA[B] long-term VA	55	[0.4854; 0.6263][0.5755; 0.8913]	Normal distributionNormal distribution	[A] vs [B]	Paired *t* test	*p* = 0.0171*
6*A*	VWT/MD(-reopening)	[A] standard VA[B] VA during MD[C] long-term VA	11/611/66	[0.5927; 0.6849][0.5339; 0.7873][0.7901; 0.9986]	Normal distributionAssumed normal distributionNormal distribution	[A] vs [B] (11/11)[B] vs [C] (6/6)[A] vs [C] (6/6)	Paired *t* testPaired *t* testPaired *t* test	*p* = 0.7291*p* = 0.0471*p = 0.0088**
6*B*	VWT/no-MD	[A] standard OPT thr[B] long-term OPT thr	55	[0.3822; 0.4110][0.3763: 0.4101]	Normal distributionAssumed normal distribution	[A] vs [B]	Paired *t* test	*p* = 0.3310
6*B*	VWT/MD	[A] standard OPT thr[B] OPT thr during MD[C] long-term OPT thr	11/611/66	[0.3831; 0.4072][0.4220; 0.4646][0.3795; 0.4363]	Normal distributionNormal distributionNormal distribution	[A] vs [B] (11/11)[B] vs [C] (6/6)[A] vs [C] (6/6)	Paired *t* testPaired *t* testPaired *t* test	**p**= 0.0002 ***p= 0.0289*p= 0.0783
6*D*	VWT/no-MD	[A] standard VA/OPT thr[B] long-term VA/OPT thr	55	NANA	NANA	VA vs OPT thrVA vs OPT thr	CorrelationCorrelation	*r* ^2^ = 0.2210*r* ^2^ = 0.2347
6*D*	VWT/MD(-reopening)	[A] standard VA/OPT thr[B] VA/OPT thr during MD[C] long-term VA/OPT thr	11116	NANANA	NANANA	VA vs OPT thrVA vs OPT thrVA vs OPT thr	CorrelationCorrelationCorrelation	*r* ^2^ = 0.0026*r* ^2^ = 0.0180*r* ^2^ = 0.0266
7*C*	VWT/no-MD	[A] standard VA[B] long-term VA	55	NANA	NANA	VA vs s/trialVA vs s/trial	CorrelationCorrelation	*r* ^2^ = 0.0741*r* ^2^ = 0.4973
7*C*	VWT/MD(-reopening)	[A] standard VA[B] VA during MD[C] long-term VA	11116	NANANA	NANANA	VA vs s/trialVA vs s/trialVA vs s/trial	CorrelationCorrelationCorrelation	*r* ^2^ = 0.1353*r* ^2^ = 0.1131*r* ^2^ = 0.0289
9*A*	VWT andSC-OI	[A] VWT/no-MD-OI[B] VWT/MD-OI[C] SC/no-MD-OI[D] SC/MD-OI	4554	[0.2754; 0.5196][-0.0677;0.1597][0.2492; 0.3908][0.04208; 0.1829]	UnknownNormal distributionNormal distributionUnknown	[A] vs [B][C] vs [D][A] vs [C][B] vs [D]	Mann–WhitneyMann–WhitneyMann–WhitneyMann–Whitney	**p**= 0.0159*p= 0.0159*p= 0.2187**p**= 0.2663
9*B*	VWT andSC-OI	[A] VWT/no-MD-OI, contra[B] VWT/no-MD-OI, ipsi[C] VWT/MD-OI, contra[D] VWT/MD-OI, ipsi[E] SC/no-MD-OI, contra[F] SC/no-MD-OI, ipsi[G] SC/MD-OI, contra[H] SC/MD-OI, ipsi	44555544	[1.649; 2.286][0.8821; 1.013][0.8455; 1.946][0.7713; 2.373][1.325; 1.915][0.7865; 1.150][0.7516; 1.878][0.9528; 1.487]	UnknownUnknownNormal distributionNormal distributionNormal distributionNormal distributionUnknownUnknown	[A] vs [B][C] vs [D][E] vs [F][G] vs [H][A] vs [C][B] vs [D][E] vs [G][F] vs [H]	Mann–WhitneyMann–WhitneyMann–WhitneyMann–WhitneyMann–WhitneyMann–WhitneyMann–WhitneyMann–Whitney	**p**= 0.0286*p= 0.8413**p**= 0.0079**p= 0.6857**p**= 0.0317*p= 0.1905**p**= 0.2857**p**= 0.0851

The table lists from left to right the figures referred to, groups compared (group), parameters analyzed (parameter), number of animals (*n*), lower (left) and upper (right) 95% confidence interval of the mean (CI_95_), distribution of the values (data structure), comparisons of (sub)groups abbreviated as indicated in the parameter column (comparison), test applied for the comparison (type of test), and statistical readout (*p*/*r* ^2^ value). OPT thr, OPT threshold; ipsi and contra, ipsilateral and contralateral eye; NA, not assessable; s/trial, seconds required per correct trial near the break. Significance levels were set as *p < 0.05; **p < 0.01; ***p < 0.001.

## Results

Essentially, we wanted to answer the following three questions. (1) Would long-term visual training (using the VWT), i.e., if visual stimuli gain behavioral importance for the mice, increase VA? (2) Would MD and extended visual training of the nondeprived eye also increase VA, as previously shown for monocular spatial frequency thresholds measured by optomotry? (3) Would long-term MD induce OD plasticity in adult mice beyond P110?

### Long-term training with a visual discrimination paradigm, the VWT, strongly increased mouse VA

To assess the effect of long-term vision training on mouse VA, SC-raised young adult mice (VWT/no-MD, *n* = 5, aged P92–P101 at the beginning of training) were trained daily in the VWT for an extended period of time (between 56 and 73 d / ≥8 weeks). Their task was to distinguish a vertical since wave grating from equiluminant gray, whereby the grating was the rewarded stimulus. After the mice had learned this task, we increased the spatial frequency of the grating until performance of the mice fell below 70% correct. After reaching similar spatial frequencies three times (standard VA) as defined by Prusky et al. (2000b), we continued training for many weeks until the animals reached five additional breaks (at the same or very similar spatial frequency values); the 3 highest of those served for the calculation of a long-term VA (for details of the VWT training, see Materials and Methods).

**Figure 2.**
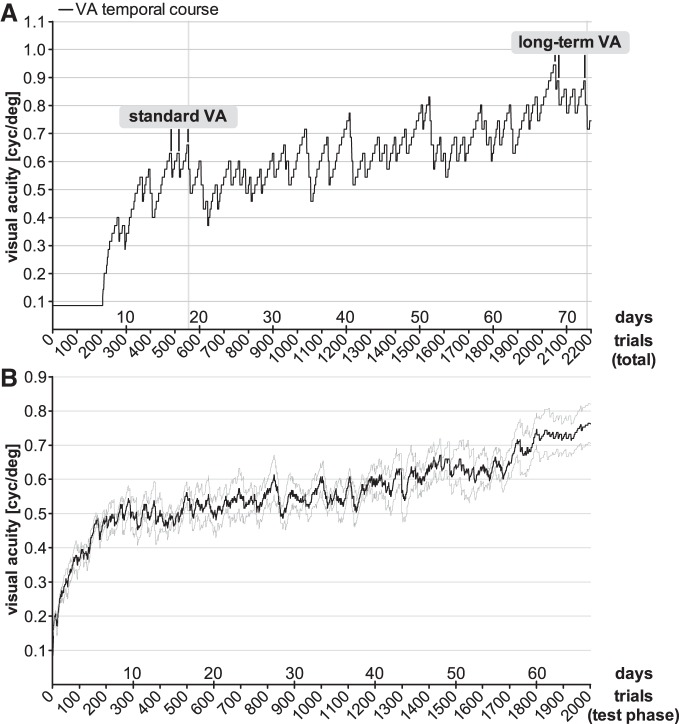
Extended VWT training strongly increased mouse VA. Mice were initially trained in the VWT to discriminate a vertical, low spatial frequency sine wave grating from isoluminant gray. Standard VA was determined after 18 ± 3 d (545 ± 88 trials) of VWT training; after a total of 66 ± 7 d (1987 ± 207 trials), long-term VA was determined. ***A***, VA of an animal in cyc/deg plotted against total trials/training days of VWT-experience. ***B***, Average VA-curve of the VWT/no-MD group (*n* = 5, mean ± SEM): individual curves were averaged starting from the test phase (time point 0). Gray vertical lines indicate the timepoints of VA and corresponding OPT threshold determination.

On average, the training necessary to obtain a standard VA for every mouse lasted 18 ± 3 d, corresponding to an actual training time of 77 ± 26 min (1 h 17 min) or 545 ± 88 trials. Long-term VA was reached after 66 ± 7 d of VWT training, corresponding to 301 ± 70 min (5 h) or 1987 ± 207 trials (Figs. 2*A*, 7). Compared to their standard VA, all animals reached significantly higher VA values: on average, VA increased from 0.56 ± 0.03 cyc/deg (standard VA) to 0.73 ± 0.06 cyc/deg (*p* = 0.017, *t* test; Fig. 6*A*, left). The average increase was 32 ± 8% (Fig. 5, left). Plots of individual VA values over time indicate that our extended VWT-training protocol allowed to determine maximal individual perceptual VA. Since values reached a plateau only after long-term VWT training (Fig. 2*A*,*B*), standard VA values apparently do not (yet) reflect individual performance thresholds, i.e., the perceptual limit of the individual mice.

Here, we also report the case of an individual mouse (mouse X) that reached a VA of 1.49 cyc/deg, a value far beyond the standard VA values reported for rats, after only 34 d (1040 trials) of VWT training (Fig. 4). Notably, the mouse followed a consistent strategy throughout the training to find the platform (Video 1). It held onto the midline divider and alternately looked at both screens repetitively before deciding where to swim. VA values steadily increased throughout the VWT training without breaks at the standard VA range. Because of this distinct behavior, we decided not to pool this mouse’s data with all other data. Training time of the mouse was 508 min (8 h 28 min) at 1040 trials, which is longer than the average time the long-term VWT group (301 ± 70 min or 5 h) spent for the complete training. Interestingly, the OPT threshold of this mouse was in a “normal” range both during (0.406/0.403 cyc/deg for the left/right eye) and after training in the VWT (0.403/0.400 cyc/deg), indicating that these two vision tasks measure different parameters of mouse spatial vision.

Video 1. Mouse X performing the VWT. In the recorded example, the rewarded visual stimulus consists of a vertical sine wave grating with a spatial frequency of 1.39 cyc/deg. The video starts with a close-up of the stimulus monitor to show the high spatial frequency of the presented grating stimulus (upper left), the nonrewarded stimulus is equiluminant gray (upper right). Reflections of the stimuli on the water surface are seen below. The mouse is released into the water-filled pool and swims toward the midline divider, holds on to it, and repeatedly looks either left or right before deciding to swim leftwards (correct response). 10.1523/ENEURO.0289-17.2017.video.1

### VA did not increase during monocular VWT training but increased after reopening the deprived eye and continued training

It has been shown previously that the spatial frequency threshold of the OPT response of the open (nondeprived) eye increases during 7 d of MD in mice and returns to pre-MD values after eye reopening when tested in the virtual-reality OPT system (Prusky et al., 2006). To test whether these findings from the virtual-reality OPT system also apply to the VWT, i.e., whether VA of the open eye would increase after MD when tested in the VWT, we tested mice before and after MD, and after reopening the previously closed eye in both vision setups: Mice were first tested binocularly in the VWT to determine their perceptual VA; on the day when standard VA was determined, we additionally determined the spatial frequency threshold of the OPT reflex of every trained mouse by optometry. Then one eye was closed in half of the mice (VWT/MD group; for details, see Materials and Methods), and the testing was continued. After determining a monocular VA in the VWT and a monocular spatial frequency threshold by optomotry, MD eyes were reopened (VWT/MD-reopening subgroup) and testing continued until a long-term VA was determined for all animals, followed by a last spatial frequency threshold measurement by optometry (see Materials and Methods).

**Figure 3.**
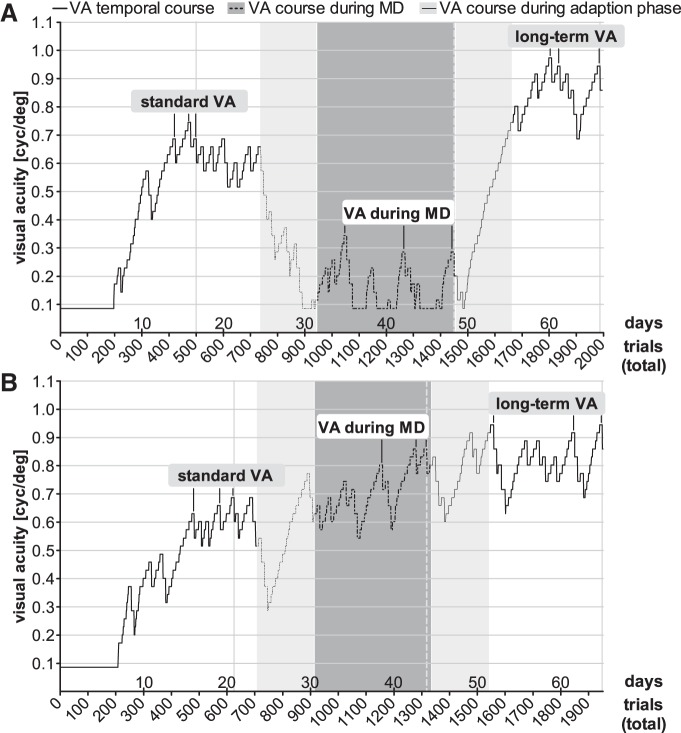
Extended monocular VWT training did not reliably increase VA, but long-term VWT training did. After determination of a standard VA, one eye was closed in animals of the VWT/MD group and mice were trained monocularly in the VWT. After an adaption phase of one week after MD (light gray background), a monocular VA (VA during MD) was determined (dark gray), then the previously closed eye was reopened, and binocular VWT training was continued (another 7 d adaption phase was introduced, light gray), until a long-term VA was determined (white). Note that individual mice (***A***, ***B***) responded quite differently to MD. Data plotted as in Figure 2*A*.

A cohort of young adult mice (VWT/MD group, *n* = 11, aged P84–P101 at the beginning of training) was trained daily in the VWT. The animals obtained MD after having achieved five breaks at the same or similar spatial frequencies. The standard VA of this group of mice before MD was 0.64 ± 0.02 cyc/deg achieved after 19 ± 3 d, corresponding to a total training time of 72 ± 29 min (1 h 12 min) or 583 ± 103 trials (Figs. 3*A*,*B*, 6*A*, right, 7).

**Figure 4.**
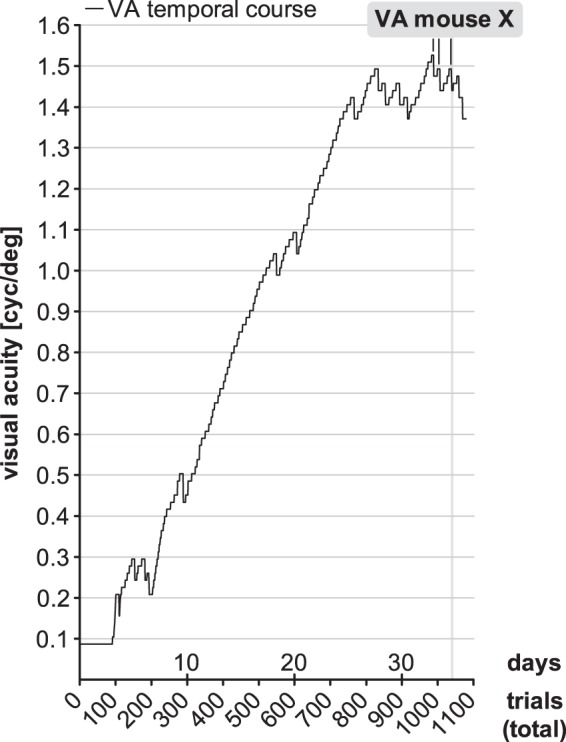
Mouse X reached a VA of 1.49 cyc/deg after 34 d of VWT training (1040 trials). This individual mouse exhibited a consistent behavioral strategy for deciding where to swim: it held onto the midline divider (see also Video 1). Data plotted as in Figure 2*A*.

**Figure 5.**
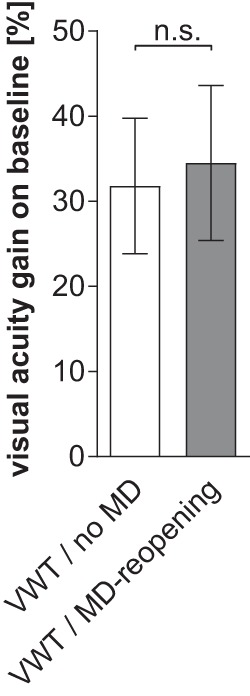
VA increase after long-term VWT training. ***A***, Gain on baseline (%) of long-term VA of the VWT/no-MD (left, *n* = 5) and VWT/MD-reopening (sub)group (MD, right, *n* = 6). Values are represented as mean ± SEM. Statistical significance was tested by a two-tailed unpaired *t* test.

**Figure 6.**
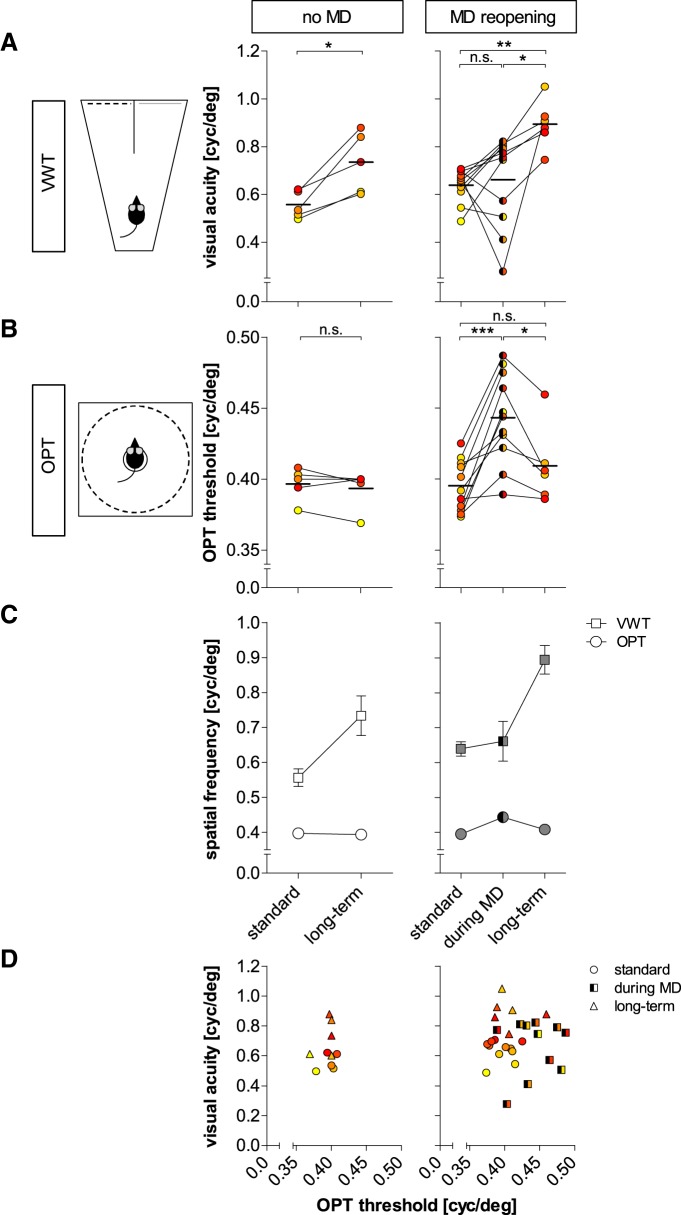
VWT and OPT thresholds of individual mice develop differently. Quantitative comparison of VWT-determined VA (***A***) and OPT thresholds (***B***) in individual animals of the VWT/no-MD group (*n* = 5) before (standard) and after long-term VWT training (left column) and in the VWT/MD group before (standard, *n* = 11), during (*n* = 11), and after MD (long-term, VWT/MD-reopening, *n* = 6/11; right column). The color code indicates low initial standard VA values in yellowish, higher values in reddish colors. ***A***, VA (cyc/deg), determined by VWT. ***B***, Spatial frequency threshold of the OPT reflex (OPT threshold, cyc/deg). ***A***, ***B***, Statistical significance was tested by two-tailed paired *t* tests; *p < 0.05; **p < 0.01; ***p < 0.001. ***C***, Average VA and OPT thresholds. Values are represented as mean ± SEM (***D***) VA plotted against OPT thresholds of individual mice.

**Figure 7.**
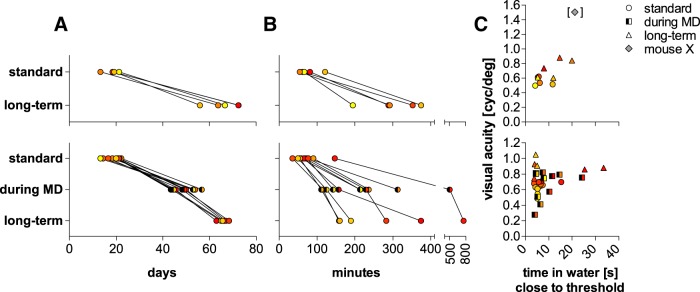
Training time in the VWT and correlation with VA. Time required to finish the different episodes of VWT training in (***A***) days and (***B***) minutes of the VWT/no-MD (upper row, *n* = 5) and VWT/MD(-reopening) group (lower row, *n* = 11 for standard and VA during MD, *n* = 6 for long-term VA after MD reopening). Individual data points are color-coded as introduced in Figure 6. ***C***, Correlation of time required per correct trial near the break (spatial frequency of the break and two frequency steps below) and VA.

**Figure 8.**
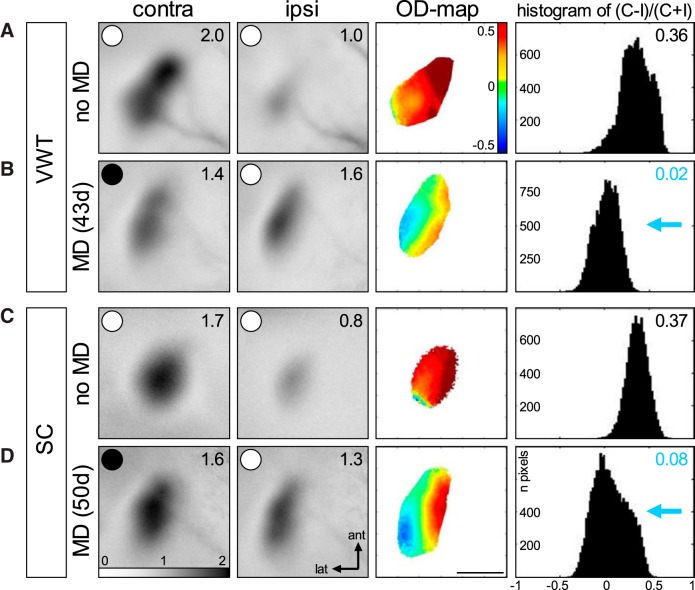
Long-term MD induced OD plasticity in adult mouse V1. Optically recorded activity maps of the contralateral (contra) and ipsilateral (ipsi) eye in the binocular part of V1 of SC-raised mice with (***A***, ***B***) and without (***C***, ***D***) VWT training, before (no-MD; ***A***, ***C***) and after MD (***B***, ***D***). Gray scale-coded activity maps (numbers correspond to quantified V1 activation), color-coded two-dimensional OD maps (color codes ODI), and the histogram of OD scores, including the average ODI, are illustrated. In mice without MD (***A***, ***C***), activity patches evoked by visual stimulation of the contralateral eye are darker than those of the ipsilateral eye, warm colors prevail in the two-dimensional OD maps and ODI values are positive. Long-term MD (MD eye is indicated by the black spot) resulted in a strong OD shift toward the open eye in both the VWT-trained and SC mice (***B***, ***D***): both eyes activated V1 more equally strong, colder colors prevailed in the OD maps, and ODI values were lower, i.e., the ODI histograms shifted to the left (blue arrows). Note that there is a visible decrease of deprived (contra) eye responses after MD in V1 of VWT mice (***B***), which is absent after MD in SC mice (***D***). ant, anterior; lat, lateral. Scale bar: 1 mm.

**Figure 9.**
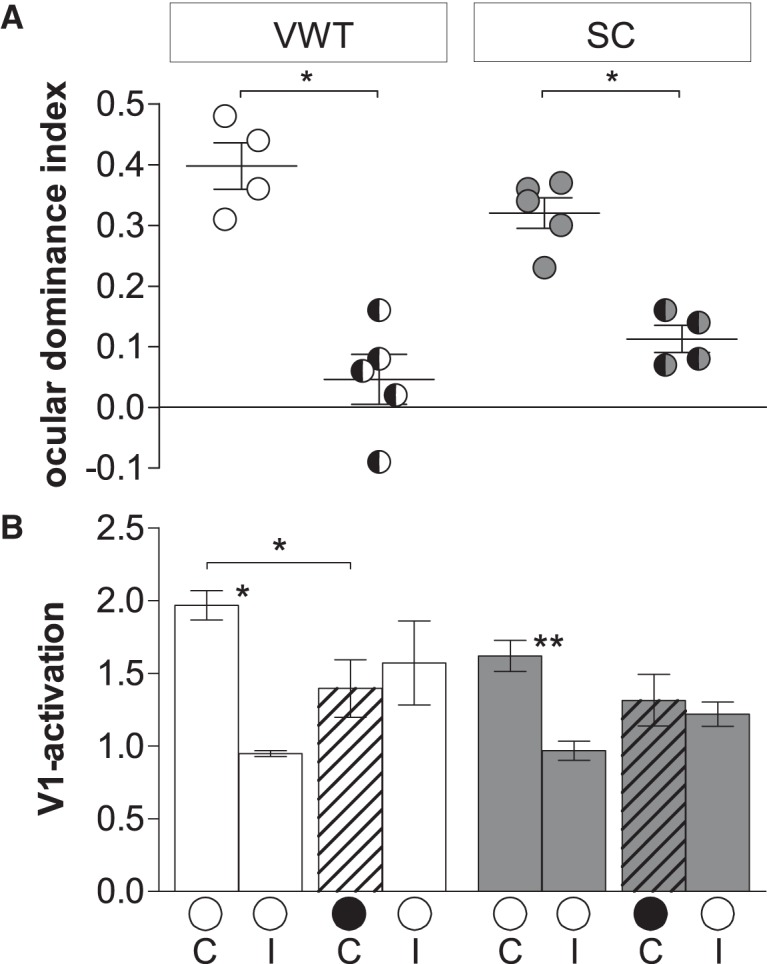
Quantification of visual cortical activation before and after MD. ODI (***A***) and V1 activation (***B***) in V1 of VWT-trained and SC mice. ***A***, Optically imaged ODIs without (no-MD) and with MD in VWT (white) and SC (gray) mice. Symbols represent ODI values of individuals; means are marked by horizontal lines. MD is indicated by half-black circles. ***B***, V1 activation elicited by stimulation of the contralateral (C) or ipsilateral (I) eye in VWT and SC mice with MD. Black filled circles indicate MD eye. ***A***, ***B***, Data represented as mean ± SEM. Statistical significance was tested by Mann–Whitney tests: *p < 0.05; **p < 0.01.

While VA of almost all animals initially dropped during the adaption phase (see Materials and Methods; Fig. 3, gray region), individual mice responded differently to the MD. In some animals, monocular VA remained strongly decreased (Fig. 3*A*); in others, monocular VA did not change or increased (Fig. 3*B*). On average, monocular VA was 0.66 ± 0.06 cyc/deg, measured after 49 ± 5 d (214 ± 114 min = 3 h 34 min) of VWT training or 1478 ± 135 trials. While individual monocular VAs fluctuated during MD (compare Figs. 3*A*,*B*, 6*A*, right), on average, values were not different from standard (binocular) VA determined before MD (*p* = 0.73, *t* test; Fig. 6*A*,*C*, right). Thus, VA of the open (nondeprived) eye did not consistently increase after MD and daily testing in the VWT, in contrast to measurements by optomotry (Prusky et al., 2006).

We next reopened the closed eye in about half of the deprived animals (VWT/MD-reopening subgroup, *n* = 6/11) to test whether binocular VA, as in the no-MD group, would further increase with long-term visual training in the VWT. This was indeed the case. All mice reached additional five breaks and after an average total training time of 66 ± 2 d (321 ± 231 min = 5 h 20 min) in 1977 ± 55 trials, long-term VA increased to 0.89 ± 0.04 cyc/deg, corresponding to an increase of 34 ± 9% compared to the standard VA of the same animals (*n* = 6, *p* < 0.01, *t* test; Figs. 5, right, 6*A*, right). Notably, the overall % VA increase was similar to that of the no-MD group (*p* = 0.83, *t* test; Fig. 5, compare left, right).

Taken together, long-term assessment of VA in the VWT resulted in significantly higher values than the standard procedure (Prusky et al., 2000b). Since the % increase of VA values of the no-MD and MD group was not different (*p* = 0.83, *t* test), we pooled values for final analysis: on average, VA increased by 33 ± 6%.

### Decision time and long-term VA do not correlate

Notably, individual mice developed different strategies for swimming toward the correct stimulus in the VWT. While some animals tended to quickly choose one path, others hesitantly and frequently floated around in front of the decision line and appeared to thoroughly scan both screens before swimming toward one of them. Sometimes animals quickly held onto the midline divider before deciding which path to take. However, we could only rarely assign one specific behavior to one specific animal. Typically, individual mice displayed a mix of different behaviors with potential tendencies for one strategy (but see mouse X). Nevertheless, it was obvious that some animals generally required more time than others to decide which path to choose, i.e., because they were holding onto the midline divider for a while. Thus, we examined whether there might be a correlation between individual VAs and the time required per correct trial near the corresponding breaks. This was not clearly the case (Fig. 7*C*): while there was no correlation for the standard VA of the no-MD group (*R* ^2^ = 0.07), there was a moderate correlation between time and long-term VA (*R* ^2^ = 0.5) of this group. Similarly, there was also no correlation between time and any of the three VAs measured in the MD group (standard VA: *R* ^2^ = 0.14; VA during MD: *R* ^2^ = 0.11, long-term VA: *R* ^2^ = 0.03), indicating that animals that required more time to decide did not generally achieve higher VA values.

Mouse X that we treated as a separate case due to its distinct (and consistent) behavior and exceptionally high VA (see above) took on average 21 s/trial near the breaks which is the longest time required compared to animals of no-MD group. However, some animals of the MD group required up to 24 s/trial for VA during MD and even 33 s/trial to achieve long-term VA, indicating that the time per trial required by mouse X cannot explain its exceptionally high VA.

### The spatial frequency threshold of the OPT reflex of the open eye increased during MD but values dissociated from VA values measured in the VWT in the same animals

Since VA values of the open eye did not reliably increase during MD despite extended vision training with the VWT (Fig. 6*A*, right), we additionally tested OPT thresholds at important time points in all VWT-trained mice. As observed previously, in mice without MD (VWT/no-MD group, *n* = 5), the spatial frequency threshold of the OPT reflex (OPT threshold) was rather constant and did not increase over time. On the day when standard VA was determined in the VWT, mice had average values of 0.40 ± 0.01 cyc/deg. Since values from left and right eye were not different (*p* = 0.85, *t* test) they were pooled for further analysis (Fig. 6*B*, left). When measured again at the end of the VWT training after the long-term VA was determined, OPT threshold was 0.39 ± 0.01 cyc/deg, and thus indistinguishable from the first measurement (*p* = 0.33, *t* test). Again, the values from both eyes were not different (*p* = 0.82, *t* test) and thus pooled.

Previous publications (Prusky et al., 2006) showed that the OPT threshold (of the open eye) increases after MD. Consistent with these findings, we also observed an increase of the pooled binocular (*n* = 11, *p* = 0.92, *t* test) OPT threshold from 0.40 ± 0.01 to 0.44 ± 0.01 cyc/deg (*n* = 11; *p* < 0.001, *t* test) after an extended MD period (34 ± 8 d), corresponding to an increase of 12 ± 2% (Fig. 6*B*, right). Thus, the OPT threshold of the open eye increased while monocular VA of the same eye tested in the VWT did not reliably increase: to allow a direct comparison of individual mouse values in both vision tests, we color-coded individual performance in Figure 6 (compare *A*, *B*) with yellowish colors indicating lower individual standard VA values, and more reddish colors indicating higher values. The same color scheme was used in Figure 7 and shows that there was no general link between training time and VA.

After reopening the previously deprived eye, OPT thresholds went back to the lower, pre-MD values (Fig. 6*B*,*C*, right), as expected from previous findings (Prusky et al., 2006). In detail, the spatial frequency threshold of the OPT reflex of the previously deprived (right) eye declined to 0.41 ± 0.01 cyc/deg (*n* = 6, *p* = 0.03, *t* test), despite continued visual training in the VWT. Values of the nondeprived (left) eye were 0.40 ± 0.01 cyc/deg. Since OPT thresholds were not different from values of the previously deprived eye (*p* = 0.12, *t* test), they were averaged to an OPT threshold of 0.41 ± 0.01 cyc/deg. Overall, the spatial frequency threshold of the OPT before MD was not different compared to the value after MD reopening and continued visual training in the VWT (*n* = 6, *p* = 0.08, *t* test; Fig. 6*B*, right).

These results clearly show that there was no correlation between OPT threshold and VA measured in the VWT (Fig. 6*D*; Fig. 6, compare *A*, *B*), neither for standard (*R* ^2^ = 0.29) or long-term VA (*R* ^2^ = 0.24) of the no-MD group, nor for standard VA (*R* ^2^ = 0.003), VA during MD (*R* ^2^ = 0.02), or long-term VA (*R* ^2^ = 0.03) of the MD group. In summary, the spatial frequency threshold of the OPT reflex of our VWT-trained mice changed in an experience-dependent way as described previously (Prusky et al., 2006): Thresholds of the open eye increased after MD and decreased back to pre-MD values after reopening of the deprived eye (Fig. 6*B*,*C*). In contrast, monocular VA of the same eye measured in the VWT did not increase after MD, but VA increased further after reopening the deprived eye and continued VWT training (Fig. 6*A*,*C*). These results clearly indicate that experience-induced increases in the spatial frequency threshold of the OPT response of the open eye do not correspond to an increased VA of the same eye/animal. In addition, our results show that values measured in the two vision setups can markedly dissociate (Fig. 6*C*). Finally, perceptual VA thresholds only increased after continued and extensive vision training in the VWT, not by MD (Fig. 6*C*,*D*, right).

### Long-term MD induced OD plasticity in adult mice independent of VWT training

In SC-raised mice, OD plasticity is age dependent: it is inducible after 4 d of MD in P25–P35 (critical period) mice; after the critical period, 7 d of MD are needed to induce an OD shift toward the open eye, and 14 d of MD failed to induce OD plasticity in animals older than P110 (Lehmann and Löwel, 2008). In contrast, various manipulations have been shown by now to prolong the sensitive phase for OD plasticity into adulthood in mice (for a recent review, see Hübener and Bonhoeffer, 2014). In particular, environmental conditions that offered increased physical activity to the mice resulted in a preserved inducibility of OD plasticity beyond P110. This raised the question whether an involuntary forced-choice visual and physical VWT training, in addition to long-term MD, can also preserve OD plasticity beyond P110.

To this end, we visualized V1 activation using intrinsic signal optical imaging in the other half of the mice (VWT/MD-OI subgroup, *n* = 5/11) that had been trained in the VWT and obtained extended MD as described above. At this point, the animals were 162-182 d old and had been monocularly deprived for 39-44 d. For control, V1 activation was additionally imaged in (1) a cohort of age-matched no-MD mice (P166–P175) that had undergone long-term VWT training (VWT/no-MD-OI group, *n* = 4, animals from results part 1), and (2) in a group of age-matched mice (P177–P180) that had obtained a duration-matched long-term MD (SC/MD-OI, *n* = 4) or no-MD (SC/no-MD-OI, *n* = 5) but were kept in SCs without access to specific physical or visual training options.

In both the VWT-trained and the SC-raised mice without MD, activation in the binocular zone of V1 was dominated by input from the contralateral eye (Fig. 8). More strongly activated brain regions require more oxygen. When illuminated with red cold light of 610 ± 10-nm wavelength, the light absorption of deoxygenated hemoglobin is higher than that of oxygenated hemoglobin, resulting in a decrease of light reflectance of active cortical regions where oxygen is transferred from blood to neurons and oxygenated hemoglobin becomes deoxygenated (Grinvald et al., 1986). Thus, activity patches after visual stimulation of the contralateral eye appear darker compared to those after ipsilateral eye stimulation (Fig. 8*A*,*C*). The relative strength of V1 activation after left and right eye stimulation was used to calculate an OD index (ODI), which is represented in a color-coded two-dimensional OD map with warm and colder colors indicating contralateral and ipsilateral dominance, respectively (Kalatsky and Stryker, 2003; Cang et al.,2005). In both VWT-trained and SC-raised nondeprived mice, OD maps were dominated by warm colors and the ODI was positive, i.e., ODI histograms peaked to the right of zero. Notably, MD resulted in an OD shift toward the open eye in both the VWT-trained (Fig. 8*B*) and SC-raised deprived mice (Fig. 8*D*), indicating that long-term MD induced OD plasticity under both conditions: the activity patches induced by ipsilateral eye stimulation now appeared as dark as those after contralateral eye stimulation, OD maps were dominated by colder colors and ODI histograms were shifted leftwards toward zero (∼0).

In detail, OD shifts were observed in adult (P162–P182) long-term VWT-trained animals after long-term MD (39–44 d) beginning after P110 (P119–P143): compared to long-term VWT-trained adult mice without MD, the ODI was reduced from 0.40 ± 0.04 (VWT/no-MD-OI, *n* = 4) to 0.05 ± 0.04 (VWT/MD-OI, *n* = 5; *p* < 0.05, Mann–Whitney test; Fig. 9*A*). Quantification of V1 activation indicates that the OD shift was mediated primarily by a reduction of deprived eye responses in V1 (Fig. 9*B*). In the nondeprived mice, V1 activation via the contralateral and ipsilateral eye was 1.97 ± 0.1 and 0.95 ± 0.02, respectively (*p* < 0.05, Mann–Whitney test). After MD, V1 activation was reduced to 1.40 ± 0.2 (*p* < 0.05, Mann–Whitney test). In contrast, while ipsilateral eye-induced V1 activation was higher than without MD, the difference was not significant (ipsilateral: 1.57 ± 0.3; *p* = 0.19, Mann–Whitney test) and similar to contralateral eye-induced V1 activation after MD (*p* = 0.84, Mann–Whitney test).

Interestingly, long-term MD (47–50 d) also induced an OD shift in the SC-raised mice that did not get long-term visual training: the ODI decreased from 0.32 ± 0.03 (SC/no-MD-OI, *n* = 4) to 0.11 ± 0.02 (SC/MD-OI, *n* = 5; *p* < 0.05, Mann–Whitney test; Fig. 9*A*). In the nondeprived mice, V1 activation following contralateral and ipsilateral eye stimulation was 1.62 ± 0.1 and 0.97 ± 0.1, respectively (*p* < 0.01, Mann–Whitney test). After MD, V1 activation after ipsilateral eye stimulation slightly increased to 1.22 ± 0.1, and V1 activation after contralateral eye stimulation slightly decreased to 1.32 ± 0.12; however, both changes were not significant (no-MD/MD: ipsilateral/contralateral, *p* = 0.09/0.29, Mann–Whitney test). Finally, V1 activation after ipsilateral and contralateral eye stimulation were not different (*p* = 0.69, Mann–Whitney test).

These findings indicate that extending the MD period to at least 39 d was sufficient to induce OD plasticity also in adult SC-raised mice.

## Discussion

Our study was aimed at working out (1) if long-term visual training, i.e., if visual stimuli gain behavioral importance, can increase mouse VA; (2) if extended monocular vision training would further increase VA, as previously shown for monocular spatial frequency thresholds measured by optomotry; and (3) if extensive and involuntary visual training during long-term MD would promote OD plasticity in adult mice beyond P110.

Here, we show that training adult SC-raised mice in a visual discrimination task, the VWT (Prusky et al., 2000b), for extended periods of time (56–73d) could indeed, and in contrast to old mice raised in a generally enriched environment (Greifzu et al., 2016), increase VA by, on average, >30%. In some animals, VA even increased to 1.1 cyc/deg, i.e., the rat range of VAs, and one mouse could see 1.49 cyc/deg, which is far beyond the rat range of VAs. Thus, if visual stimuli get behaviorally important (e.g., to avoid excessive swimming) mouse spatial vision can dramatically improve and visual acuity can nearly double in individual animals. A recent study investigating the effects of visual training with various stimuli on mouse VA supports our observations. Wang et al. (2016) observed VA increases of 55% after a total of 48–49 d of VWT training (subdivided in 8 to 9 d pretraining VA, 35 d training, 4 d post-training VA assessment). At first glance, this may indicate stronger VA increases in the Wang et al. (2016) study compared to our present results. However, the seemingly stronger VA increase of the Wang et al. (2016) study was entirely caused by significantly lower pretraining VA values of around 0.46 cyc/deg, obtained after only 8–9 d of VWT training. In contrast, our average baseline VA values were 0.56-0.64 cyc/deg, determined after 18–19 d of VWT training. The maximal (long-term) VA values obtained in the present study were 0.74 for the VWT/no-MD group and 0.89 cyc/deg for the VWT/MD-reopening subgroup, achieved after 56–73 d of VWT training. These values are slightly higher than the 0.74 cyc/deg, measured after 48–49 d of VWT training, reported by Wang et al. (2016). Individual animals’ performance curves of our study (Fig. 2*A*) illustrate that visual acuities reached a plateau after long-term testing, indicating that indeed the maximum VA, i.e., the perceptual threshold was determined. VA measured by Wang et al. (2016) must not necessarily have reached a plateau yet so that VA of their mice could potentially have further increased by continued training (see Wang et al., 2016, their Fig. 2*A*,*D*). Taken together, both studies agree that mouse perceptual VA can increase dramatically if vision gets behaviorally important, and maximal VA values are only reached after extended behavioral vision training. It would be very interesting to measure wild mouse VA to test whether the generally restricted environments of laboratory mice are causing low VAs or whether only special vision training can increase thresholds.

Notably, the mouse with the extremely high VA of nearly 1.5 cyc/deg (mouse X) displayed a consistent behavior in the VWT before deciding where to swim by holding onto the midline divider and was thus taking a long time (21 s) to reach a decision, which might have been important for reaching a high VA. However, correlating the time a mouse spent in water per correct trial near the corresponding breaks did not yield a consistent correlation for all tested animals. While there was a weak correlation between time and long-term VA of the no-MD group, there was no correlation for the MD group. Furthermore, there were mice in our sample that spend on average up to 33 s and had VA values in the order of 0.8 cyc/deg. Thus, we cannot attribute the high VA of mouse X to the time spent for decision-making. We therefore conclude that the distinct behavior of mouse X was most likely not causally related to its extremely high VA.

Would extended monocular training also increase VA of the trained, open eye? The answer is a clear “no.” To allow a direct comparison of measured values in both vision tests in individual mice, we tested the spatial frequency threshold of the OPT response before MD, during MD and after MD-reopening and additional long-term training. All OPT-measurements were performed on the same days when VA (standard VA, monocular VA, long-term VA) was assessed using the VWT in individual animals. Consistent with the literature (Prusky et al., 2006), OPT thresholds of the open eye increased after MD. After MD-reopening, thresholds again decreased and were similar to initial pre-MD values. In contrast to OPT thresholds, monocular VA using the VWT did not reliably increase during MD. While values of individual mice were very variable, on average, VA values after MD were not different from pre-MD values. After reopening of the previously deprived eye and continued VWT training, VAs increased even further while at the same time and in the same animals, OPT thresholds decreased.

Thus, using the VWT, instead of returning to pre-MD standard VA values, VA increased further after reopening the MD eye, and values were independent of the VA observed during MD. Additionally, the increase in VA when comparing standard with long-term VA was present in both the VWT/MD-reopening and the VWT/no-MD animals, indicating that MD did not contribute to the overall increase in VA.

This conclusion is supported by the observation that there was also no significant difference in VA-improvement between binocularly trained mice and the trained eye of monocularly trained mice (Wang et al., 2016). Thus, long-term training in general rather than forced monocular vision effectuated a significant increase in behaviorally measured VA.

Our detailed comparisons of quantified visual parameters in the two most commonly used mouse vision tests, VWT and optomotry, thus clearly revealed that values are independent of each other and can completely dissociate. The two tests obviously measure completely different parameters of mouse spatial vision. This further indicates that not MD, but extended visual training is the driving force to increase mouse VA.

The observed discrepancy between the used methods raises questions regarding different aspects of vision being measured by each test. While the OPT response is mediated by the brainstem accessory optic system, cortical circuits have been shown to be important for the experience-dependent increase in OPT thresholds after MD (Prusky et al., 2006). Notably, the highest reported values of mouse OPT thresholds after MD are 0.54-0.55 cyc/deg (Prusky et al., 2006; Lehmann and Löwel, 2008) and thus in the range of baseline VA values determined in the VWT (Prusky et al., 2000b) that presumably exploit V1-processing machinery. The results of the present study support this correlation: individual OPT thresholds of the open eye at the end of the MD period were usually similar or slightly lower than VWT-determined VAs of the same animal. Additionally, OPT thresholds of mouse X were in the normal range. Measurements in the VWT require associative learning and cortical processing. Since it was recently demonstrated that cortico-fugal projections from V1 to the accessory optic system, in particular to the optic tract and dorsal-terminal nuclei (NOT-DTN), underlie learning-induced optokinetic reflex potentiation (Liu et al., 2016), an experience-dependent recruitment of V1 may also underlie the experience-dependent enhancement of OPT thresholds after MD into the range of the generally higher “cortical” VA. Another recent paper clearly showed that V1 had a key role in discrimination learning underlying our VA determination (Jurjut et al., 2017).

There may also be differences with respect to the part of the visual field used for the two vision tests. As shown before (Prusky et al., 2006), OPT enhancements are restricted to the monocular visual field while visual stimuli in the VWT are presented in the binocular visual field. In fact, the strong reductions in VWT-measured VA after MD that we observed in some of our mice could be explained by assuming that these mice performed the VWT using binocular interactions, so that their performance degraded when only one eye was available for vision. Alternatively, some mice could have a preferred eye for performing the VWT, and if their preferred eye was closed, performance would also be expected to degrade. However, VA of most mice did not decrease after MD. additional experiments are needed to clarify these issues and to determine if mice use true binocular fusion in the VWT or use input from one eye only.

Taken together, the observed different development of VA (measured via VWT) and OPT threshold (measured via optomotry) during MD is therefore likely a result of different neuronal circuits involved, different visual capabilities assessed and different involvement of information processing entities in the CNS owing to a different complexity of the tasks.

There is consensus in the literature that OD plasticity is age dependent in SC-raised mice, with clearly decreasing plasticity in adult animals beyond P110, even if MD is prolonged to two weeks (Lehmann and Löwel, 2008; Sato and Stryker, 2008), although there are reports of OD plasticity in even older animals (Sawtell et al., 2003; Pham et al., 2004; Hofer et al., 2006). In recent years, numerous environmental and behavioral interventions have been reported that can restore plasticity in adult or old SC-raised rodents or sustain the potential to induce plastic changes in adult rodent V1 beyond that age (Espinosa and Stryker, 2012; Hübener and Bonhoeffer, 2014). For example, previous MD (Hofer et al., 2006), forced visual stimulation (Matthies et al., 2013), visual stimulation and running (Kaneko and Stryker, 2014), and dark rearing (He et al., 2006; Stodieck et al., 2014) promoted V1 plasticity in adult rodents. In particular, mice or rats that had access to a running wheel or were raised in a generally enriched environment remained susceptible to OD plasticity into late adulthood (Sale et al., 2007; Baroncelli et al., 2010; Greifzu et al., 2014), proving that voluntary physical exercise can promote the brain’s potential to adapt to environmental changes. While raising mice in an enriched environment can even support a lifelong OD plasticity in mice, it did not improve visual abilities of old animals (Greifzu et al., 2016). Here, in contrast, we demonstrate that physically exercising in a visually demanding, complex task clearly resulted in improved VA. We furthermore show that extended physical exercise is, however, not necessary for promoting OD plasticity after long-term MD in V1 of adult mice. Surprisingly, OD plasticity was not only induced in mice trained in the VWT during MD, i.e., in the mice in which vision gained behavioral importance, but also in age- and MD duration-matched SC-raised mice without VWT training, proving that a longer-term MD (at least 39 d) was sufficient to induce OD plasticity in adult mice (beyond P110). This result is noteworthy, because in mice up to P91, 7-8 d of MD had already a saturating effect on the OD, and longer MD duration did not make OD shifts stronger (13-22 d; Sato and Stryker, 2008). In contrast, our new data clearly show that longer-term MD can indeed induce OD shifts in SC mice up to at least P182. Furthermore, our new results indicate that SC raising may dramatically slow down plastic changes of brain circuits, and that these changes can be promoted or sped up by interventions such as those mentioned above, including extended vision training and physical exercise (Kalogeraki et al., 2017). Interestingly, however, there were some differences in the OD plasticity between the VWT-trained versus nontrained SC mouse group: OD shifts were slightly larger in the VWT-trained mice [ODI difference of 0.35 (VWT) vs 0.21 (SC) comparing ODIs before and after MD], and additionally, there was some evidence that the mechanism underlying the plastic changes may be different. Although the differences were not pronounced, statistical testing revealed that OD shifts in the VWT-trained SC mice, i.e., mice with intensive visual and physical training, were primarily mediated by a decrease of deprived eye responses in V1. While a weakening of deprived eye responses in V1 is characteristic for the juvenile form of OD plasticity, and is rapidly induced during the critical period (P20–P35) by 4 d of MD (Espinosa and Stryker, 2012) delayed open eye strengthening becomes the dominant (Frenkel and Bear, 2004; Tagawa et al., 2005) or sole process mediating OD shifts in young adult SC mice after 7 d of MD (Sawtell et al., 2003; Hofer et al., 2006; Sato and Stryker, 2008; Ranson et al., 2012). Additional experiments are needed to clarify whether additional VWT training indeed contributes to a more juvenile form of OD plasticity induced by long-term MD in adult mice as compared to SC housing during MD.

It is worth noting that the VWT/MD-reopening subgroup showed an increase in VA after eye reopening, although MD might have weakened V1 activation via the (previously) deprived eye, indicating that OD is not a correlate of VA, i.e., does not reliably predict the behaviorally measurable change in visual performance.

Together, these data indicate that there is most likely a trade-off between age and MD duration in SC-raised animals: in younger mice, shorter MDs are sufficient to induce significant OD shifts. In contrast, older SC-raised mice need considerably extended MD duration to display OD plasticity. These long MD times can be shortened after specific environmental and behavioral interventions such as, e.g., previous MD at young ages (Hofer et al., 2006), raising animals in an enriched environment (Sale et al., 2007; Greifzu et al., 2014), voluntary running (Kalogeraki et al., 2014), forced visual stimulation (Matthies et al., 2013), and dark exposure (He et al., 2006; Stodieck et al., 2014).

Summarizing, our data clearly show that mouse VA can increase dramatically if visual stimuli gain behavioral importance. Individual animals can even reach VA values in and even beyond the rat range of VAs. In contrast to OPT measurements, that show increased open eye spatial frequency thresholds after MD, monocular VA of the same mice did not increase in the VWT. However, VA increased after reopening of the previously deprived eye and continued VWT training. The comparison of optomotry and VWT-measured values thus further indicates that the two commonly used mouse vision tests measure different parameters of mouse spatial vision, and that quantified values can totally dissociate. Finally, and surprisingly, long-term MD can induce OD plasticity also in SC-raised adult mice (≥P177), and there were some indications that OD shifts in VWT-trained SC mice might be mediated primarily by reductions in deprived eye responses in V1.

## Synthesis

Reviewing Editor: Tatyana Sharpee, The Salk Institute for Biological Studies Decisions are customarily a result of the Reviewing Editor and the peer reviewers coming together and discussing their recommendations until a consensus is reached. When revisions are invited, a fact-based synthesis statement explaining their decision and outlining what is needed to prepare a revision will be listed below. The following reviewer(s) agreed to reveal their identity: Lynne Kiorpes, Daniel Tso.

The manuscript presents interesting results about the impact of training on adult cortical plasticity and monocular deprivation, an important field of high interest. Reviwers make specific suggestions for improving readability of the manuscripts, clarifying methodological detail, and resolving apparent statistical discrepancies in Figure 9.

Reviewer 1:

Optical imaging assessment of ocular dominance shifts following the period of MD highlights an important point (which the authors should discuss) that ocular dominance is not an index - or even a correlate - of acuity.

The comparison of the two assessment methods is interesting because it highlights that the method used matters. The conflicting results from the two methods are not that surprising. Optomotor responses are not in general an index of cortical function. One presumes that even in rodents behaviorally measured acuity relies on cortical function. It would be quite unlikely that acuity would improve dramatically and then return back to pre-MD levels immediately, within a short period of MD. There is definitely value in the fact that this study shows the data from two methods taken at the same time points from the same animals, and that the behavioral measure shows the expected improvement with extensive experience.

The section on optical measures of ocular dominance from the MD is interesting because it shows that the training itself had no particular impact on that outcome. The OD shift was the same in the trained and non-trained animals. The subtle distinction the authors make between the two groups in terms of 'open eye increase' and 'deprived eye decrease' is completely unconvincing (the open eye comparison shows a larger, but non-significant difference for the trained group compared to the non-trained group which shows what apparently is a significant difference). Either the bar graph is wrong (Fig. 9B) or the statistics are wrong. The very small, unequal n in these groups could produce a spurious result.

In general, the manuscript needs cleaning up and the methods need to be clarified. Training durations, durations of MD, and the like are not made clear. The rule for stopping training is very odd - based on “breaks” - I'm not sure I understand what they did. Numbers of animals and ages are only stated in the text of the Results section. They state that the amount of training for the MD and non-MD groups is the same but given the described method and stopping rule that can't be the case. So there is some clarification and consistency needed.

Reviewer 2

This manuscript describes experiments examining the impact of long-term visual training and long-term monocular deprivation (MD) on the visual acuity of adult mice. The results are interesting and add to the literature on adult cortical plasticity (beyond the critical period). However the manuscript could be shortened and improved for readability. There are several portions of text that are repetitious and are minor rephrasing of other portions of the manuscript. The Discussion section also excessively restates the results and otherwise focuses on descriptive synthesis and analysis rather than exploring possible mechanisms, etc.

One of the most striking findings presented concerns the differences between the improvements in visual acuity (VA) using the visual water task (VWT) as compared with VA as determined by optomotry (spatial frequency threshold). Yet the authors write little about the possible meaning or interpretations of this marked difference.

The authors spend substantial text describing the unique results of one individual mouse (X) that exhibited exceptional performance and therefore its results were not pooled with the rest. The significance of those outlier findings is difficult to assess.

Its unfortunate that the authors did not devise a means to test VA monocularly using VWT (post-MD), as it would be helpful in interpreting the impact of MD on each of the eyes separately. Similarly, in the presumptive binocular testing, it would seem to be possible that the mouse only uses the input from one eye instead of true binocular fusion (no binocular enhancement effect is demonstrated).

The variability in the behavior (and performance) during MD in different individual mice (Figures 3, 6), may indicate that some aspect of the MD procedure or surgery that is unrelated to neural plasticity in the visual system is contributing to the overall behavior (pain? irritation? distraction?) that may in turn taint the MD results.

The authors show that both VWT and SC mice subjected to long-term MD exhibited OD plasticity similarly, but this result of MD has largely been reported previously (e.g. Hofer et al, 2006; Sato and Stryker, 2008). The authors claim the VWT had “at least some rejuvenating effect”, and some differences between the two classes, but these differences appears to be quite weak.
